# President's Address

**Published:** 1903-08-08

**Authors:** 


### The British Medical Association.

### PRESIDENT'S ADDRESS.

In delivering the opening address, Dr. Griffiths, the President, chose for his theme the evolution of antiseptic surgery and its influence on the progress and advancement of bacteriology and therapeutics. He commenced by giving a picture of what surgery used to be like some forty years ago in the best London hospitals. In the latter end of the fifties and early sixties the large wards of our hospitals were filled with all sorts of cases indiscriminately—simple and compound fractures, suppurating joints, traumatic, senile, and hospital gangrene, erysipelas, septicæmia, pyæmia, and cases operated on for accidents and diseases, all of which were dressed by the same attendants. Each ward had its set of sponges for the purpose of dressing wounds, and these were used indiscriminately for the various cases under treatment. There was no such thing as absorbent cotton wool in those days, and great economy was observed in the use of lint. Linseed meal and charcoal poultices were frequently employed to sweeten and cleanse wounds and to encourage healthy suppuration. "Laudable pus" was a sign of good dressing and healthy action.

The operating theatre was often crowded with students from the dissecting-room, and the surgeon was in the habit of wearing an old black frock coat, kept for the purpose, and well stained with blood and matter from previous cases. The same sponges which were used in the wards were also used in the operating theatre. Such was the condition of surgery previous to the publication by Lister in 1867 of the principles of antisepsis. Naturally enough the practical application of Lister's principles has been gradually modified and improved. The use of the spray has been given up with the result that more attention is now paid to the purity of the atmosphere in the wards and operating theatres. This has led to a consideration of the possibility of asepticism, with the view of discarding antiseptics at operations and for the dressing of wounds. When no antiseptic lotion or swabs are used, the aërial micro-organisms settling in the wound are left to the digestive cells and phagocytes to be disposed of. In ordinary circumstances and with proper aseptic precautions they are able to do so; but it cannot be denied that this delegation of trust to the cells, coupled with the possibility of imperfect sterilisation of the skin and materials used at the operation, involves greater risk of disasters than when antiseptic means are also used. Care must be taken that the pendulum does not swing too far to the aseptic side. The result of Lister's antiseptic surgery has been to reduce the mortality from major operations from about 33 per cent., which it was 36 years ago, to about 3 per cent.

The evolution of antiseptic surgery, based as it was on the discoveries of Schwann and Pasteur, gave a great impetus to the study of bacteriology, with the result that new and unexpected discoveries were made as to the part played by bacteria in nature. And indirectly our knowledge of the action of medicines was materially advanced. Thus quinine, whose beneficial properties in the treatment of malaria were discovered by accident, is now recognised to act simply as a germicide, destroying the malarial parasites introduced into the blood by mosquitos. And so with many of the most valuable and most frequently used drugs in use; their beneficent action may reasonably be thought to be due partly or wholly to their power of destroying micro-organisms. Thus salicin, salicylic acid, and the salicylates are powerful antiseptics, so also are mercurial salts, arsenic, and iodine.

The local treatment of skin diseases, as well as those of the mucous membranes, might be said to consist in a judicious use of antiseptics alone, or in combination with some sedative or stimulating agent. From these facts it will be seen that Listerism is as valuable in the practice of medicine as it is in surgery.

The practical application of our knowledge for the benefit of the public health has not kept pace with the great progress made in medicine and surgery, and the fault of this lies in the want of better legislation. Public health officers should be appointed to large districts solely for public health duties, and they should be excluded from private practice. The service should be sufficiently tempting to attract some of the best talent in the profession. The appointment, in the first place, should be competitive, the tenure of office secure, and there should be provision for superannuation. At the present time, although every rural sanitary authority is required to appoint a medical officer of health, the duties and salary of the officer are left to the discretion of the local authority, except in those cases in which part of his salary is repaid by the Local Government Board. Rural sanitary authorities are generally satisfied with very little more than the exercise of their power of electing their officer and the fixing of a small salary. The officer, who is as a rule a local practitioner, is allowed to limit his own work as he may think proper; and as the tenure of his appointment is precarious, he is naturally disposed, in the performance of his duties, to interfere as little as possible with individual interests; practically he has no power to correct matters which come under his notice. Dr. Griffiths estimated that a moderate improvement of our sanitary administration would reduce the annual death rate by 1·5 per 1,000 living. Supposing the population of the United Kingdom to be 40,000,000, the saving of life would thus amount to 60,000 a year.

